# CT-based Visual Classification of Emphysema: Association with Mortality in the COPDGene Study

**DOI:** 10.1148/radiol.2018172294

**Published:** 2018-05-15

**Authors:** David A. Lynch, Camille M. Moore, Carla Wilson, Dipti Nevrekar, Theodore Jennermann, Stephen M. Humphries, John H. M. Austin, Philippe A. Grenier, Hans-Ulrich Kauczor, MeiLan K. Han, Elizabeth A. Regan, Barry J. Make, Russell P. Bowler, Terri H. Beaty, Douglas Curran-Everett, John E. Hokanson, Jeffrey L. Curtis, Edwin K. Silverman, James D. Crapo, For the Genetic Epidemiology of COPD (COPDGene) Investigators

**Affiliations:** From the Department of Radiology (D.A.L., D.N., T.J., S.M.H.), Division of Biostatistics (C.M.M., C.W., D.C.E.), and Department of Medicine (E.A.R., B.J.M., R.P.B., J.D.C.), National Jewish Health, 1400 Jackson St, Denver, CO 80206; Department of Radiology, Columbia University Medical Center, New York, NY (J.H.M.A.); Department of Diagnostic Radiology, Hôpital Pitié-Salpêtrière, Assistance Publique–Hôpitaux de Paris, Sorbonne Universités, Paris, France (P.A.G.); Department of Diagnostic and Interventional Radiology, University of Heidelberg, Translational Lung Research Center Heidelberg, Heidelberg, Germany (H.U.K.); Division of Pulmonary and Critical Care Medicine, Department of Internal Medicine, University of Michigan Health System, Ann Arbor, Mich (M.K.H., J.L.C.); Department of Epidemiology, Johns Hopkins Bloomberg School of Public Health, Baltimore, Md (T.H.B.); Department of Epidemiology, Colorado School of Public Health, University of Colorado Denver, Anschutz Medical Campus, Aurora, Colo (J.E.H.); Medical Service, VA Ann Arbor Healthcare System, Ann Arbor, Mich (J.L.C.); and Division of Network Medicine, Brigham and Women’s Hospital and Harvard Medical School, Boston, Mass (E.K.S.).

## Abstract

**Purpose:**

To determine whether visually assessed patterns of emphysema at CT might provide a simple assessment of mortality risk among cigarette smokers.

**Materials and Methods:**

Of the first 4000 cigarette smokers consecutively enrolled between 2007 and 2011 in this COPDGene study, 3171 had data available for both visual emphysema CT scores and survival. Each CT scan was retrospectively visually scored by two analysts using the Fleischner Society classification system. Severity of emphysema was also evaluated quantitatively by using percentage lung volume occupied by low-attenuation areas (voxels with attenuation of −950 HU or less) (LAA-950). Median duration of follow-up was 7.4 years. Regression analysis for the relationship between imaging patterns and survival was based on the Cox proportional hazards model, with adjustment for age, race, sex, height, weight, pack-years of cigarette smoking, current smoking status, educational level, LAA-950, and (in a second model) forced expiratory volume in 1 second (FEV_1_).

**Results:**

Observer agreement in visual scoring was good (weighted κ values, 0.71–0.80). There were 519 deaths in the study cohort. Compared with subjects who did not have visible emphysema, mortality was greater in those with any grade of emphysema beyond trace (adjusted hazard ratios, 1.7, 2.5, 5.0, and 4.1, respectively, for mild centrilobular emphysema, moderate centrilobular emphysema, confluent emphysema, and advanced destructive emphysema, *P* < .001). This increased mortality generally persisted after adjusting for LAA-950.

**Conclusion:**

The visual presence and severity of emphysema is associated with significantly increased mortality risk, independent of the quantitative severity of emphysema.

*Online supplemental material is available for this article.*

## Introduction

Chronic obstructive pulmonary disease (COPD) is the third most common cause of death in the US, accounting for 5.6% of all deaths in 2014 (1). Factors known to be associated with increased mortality from COPD include severity of airflow obstruction, body mass index, dyspnea, exercise capacity, and quantitative severity of emphysema (2–4). Although COPD is a convenient clinical label with a clear physiologic definition, pathologic and CT evaluations show that it is a heterogeneous group of disorders, comprising a range of patterns of emphysema, chronic bronchitis, and nonemphysematous obstruction due to small-airway disease that vary among individuals (5). Importantly, individuals with similar levels of physiologic impairment may have very different CT appearances. Additionally, cigarette smokers who do not have COPD can have emphysema (6). CT has been extensively validated as a tool for assessment of the presence, pattern, and severity of emphysema (7–10). Quantitative CT evaluation can successfully identify emphysema, expiratory airflow obstruction, and airway wall thickening (11), but has not been shown to fully capture the information available from visual subtyping of emphysema. Visual and quantitative CT evaluation are currently regarded as complementary methods to assess COPD (12).

Centrilobular emphysema (CLE) is the prototypical form of emphysema identified in cigarette smokers (13,14), while paraseptal emphysema is also clearly smoking related (15,16). A recently published visual classification system from the Fleischner Society grades the severity of parenchymal (nonparaseptal) emphysema as trace, mild, moderate, confluent, and advanced destructive emphysema (12). Because true panlobular emphysema seems to be uncommon in smoking-related emphysema, this classification applies the terms confluent emphysema and advanced destructive emphysema to what previously was called panlobular emphysema, and the term panlobular emphysema is now reserved for the emphysema found in subjects with α-1 antitrypsin deficiency. Using this system in 1540 subjects enrolled in the COPDGene study, we showed a genome-wide significant association with visual severity of parenchymal emphysema at the 15q25 region (*P* = 6.3e-9) (17).

There has, to our knowledge, been no previous analysis of the relationship between visually assessed emphysema pattern and mortality. We had the opportunity to apply this grading system in a large population of cigarette smokers enrolled in the COPDGene study, who underwent thin-section chest CT and have now been followed for more than 5 years. We hypothesized that more severe grades of parenchymal emphysema would be associated with higher mortality, even after adjustment for other important covariates. The purpose of our study was to evaluate the relationship between visually assessed CT abnormality and mortality.

## Materials and Methods

COPDGene is a prospective and multicenter investigation focused on the genetic epidemiology of COPD (ClinicalTrials.gov: NCT00608764) (18). Between 2008 and 2011, 10 192 cigarette smokers were enrolled in our Health Insurance Portability and Accountability Act–compliant study at 21 centers in the United States. Institutional review board approval of the research protocol was obtained at all clinical centers, and written informed consent was obtained from all participants. The project described was supported by Award Number U01 HL089897 and Award Number U01 HL089856 from the National Heart, Lung, and Blood Institute. The COPDGene project is also supported by the COPD Foundation through contributions made to an Industry Advisory Board composed of AstraZeneca, Boehringer Ingelheim, GlaxoSmithKline, Novartis, Pfizer, Siemens, and Sunovion.

Participants were all current or former smokers with at least 10 pack-years of exposure to smoking. All subjects self-identified as either non-Hispanic African American or non-Hispanic White. Subjects with respiratory conditions other than asthma and COPD were excluded. For this report, we evaluated quantitative and visual analysis of emphysema on the baseline CT scans of the first 4000 subjects consecutively enrolled in COPDGene. The first 4000 were chosen because the duration of follow-up of this group would be longer, and because visual analysis of the remainder of the cohort was not yet complete. There were 829 subjects excluded, most commonly because mortality ascertainment was not adequate (Fig E1 [online]), resulting in our final study population of 3171 participants. The study population comprised 1690 men and 1481 women, with a mean age of 60.4 years ± 9.2 (standard deviation) (60.2 years ± 9.2 for men, and 60.6 years ± 9.2 for women) and an age range of 41.5–85.0 years. Compared with subjects retained for analysis, the excluded subjects were slightly younger, more likely to be male, African American, and current smokers, but showed similar levels of symptomatic and functional impairment (Table E1 [online]).

### Clinical Evaluation

On enrollment, all subjects underwent spirometry, evaluation of bronchodilator responsiveness and 6-minute walk test using standard techniques (18). Standardized questionnaires were used to evaluate respiratory symptoms (St George Respiratory Questionnaire [SGRQ]) (19), dyspnea score (modified Medical Research Council [MMRC] dyspnea score [20]), history of exacerbations and symptoms of chronic bronchitis. Comorbid diseases (including coronary artery disease, congestive heart failure, and diabetes) were identified on questionnaire at the time of enrollment, based on self-report of physician diagnosis. The BODE (body mass index [BMI], degree of airflow obstruction, dyspnea, and exercise capacity) index, a predictive index of mortality in COPD, was calculated from clinical parameters (21). The severity of airflow obstruction was classified according to the Global Initiative for Obstructive Lung Disease (GOLD) stages (22), including the newly recognized Preserved Ratio Impaired Spirometry (PRISm) group, where FEV_1_ is reduced but the ratio of FEV_1_ to forced vital capacity (FVC) is decreased (23,24).

### Quantitative CT Analysis

All subjects underwent volumetric inspiratory and expiratory CT using a standardized protocol (18,25,26). Anonymized scans were transferred to a central imaging laboratory at our institution for visual and quantitative analysis. We used dedicated software programs to perform quantitative analysis of the severity of emphysema (3DSlicer; *http://www.slicer.org*), (Pulmonary Workstation 2; Vida Diagnostics, Coralville, Iowa) (26).

### Visual Analysis

Visual analysis by trained research analysts was based on the Fleischner Society classification system (12) (Fig 1). The analysts had no previous experience in radiologic interpretation. Detailed methods are provided in Appendix E1 (online).

**Figure 1a:**
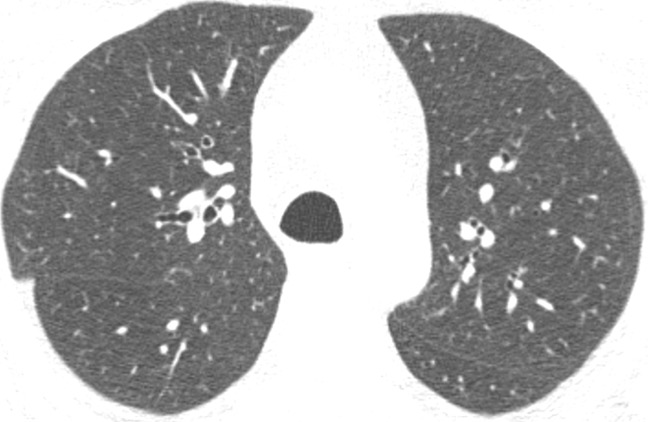
Axial CT images show severity grades of parenchymal emphysema. **(a)** Normal CT scan shows no emphysema. **(b)** Image shows trace centrilobular emphysema (circle), which involved less than 0.5% of the lung zone. **(c)** Image shows mild centrilobular emphysema (arrows), which involved 0.5%–5.0% of the lung zone. **(d)** Image shows moderate centrilobular emphysema, which involved more than 5% of the lung zone. **(e)** Confluent emphysema. **(f)** Advanced destructive emphysema with vascular distortion.

**Figure 1b:**
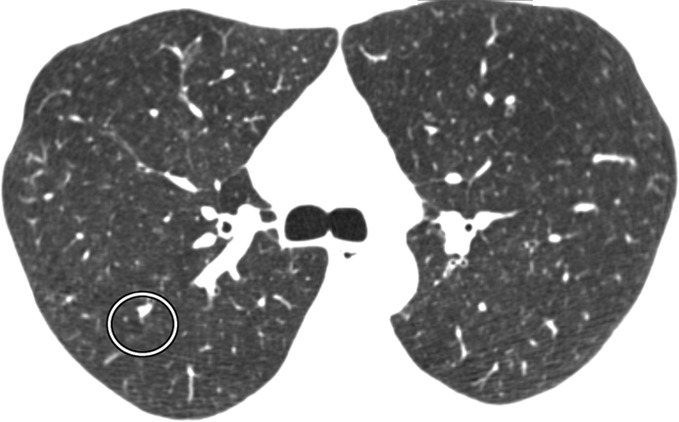
Axial CT images show severity grades of parenchymal emphysema. **(a)** Normal CT scan shows no emphysema. **(b)** Image shows trace centrilobular emphysema (circle), which involved less than 0.5% of the lung zone. **(c)** Image shows mild centrilobular emphysema (arrows), which involved 0.5%–5.0% of the lung zone. **(d)** Image shows moderate centrilobular emphysema, which involved more than 5% of the lung zone. **(e)** Confluent emphysema. **(f)** Advanced destructive emphysema with vascular distortion.

**Figure 1c:**
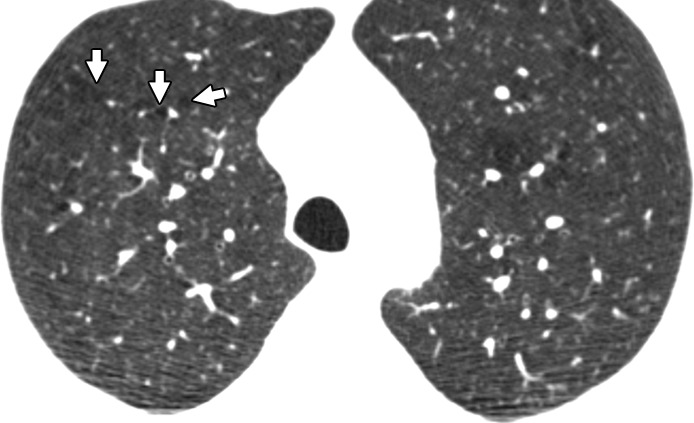
Axial CT images show severity grades of parenchymal emphysema. **(a)** Normal CT scan shows no emphysema. **(b)** Image shows trace centrilobular emphysema (circle), which involved less than 0.5% of the lung zone. **(c)** Image shows mild centrilobular emphysema (arrows), which involved 0.5%–5.0% of the lung zone. **(d)** Image shows moderate centrilobular emphysema, which involved more than 5% of the lung zone. **(e)** Confluent emphysema. **(f)** Advanced destructive emphysema with vascular distortion.

**Figure 1d:**
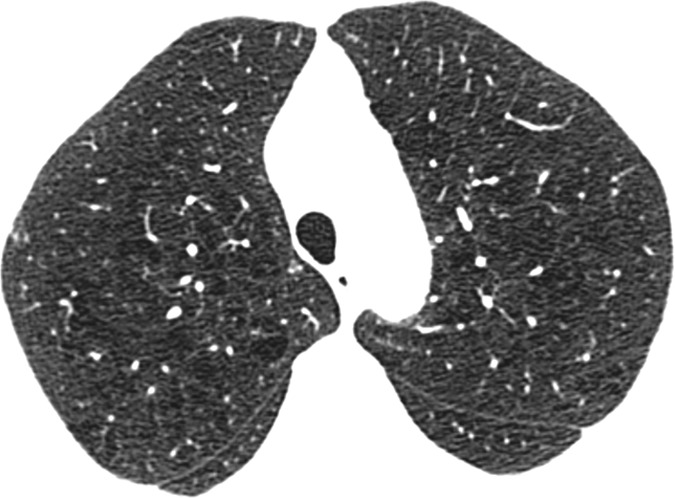
Axial CT images show severity grades of parenchymal emphysema. **(a)** Normal CT scan shows no emphysema. **(b)** Image shows trace centrilobular emphysema (circle), which involved less than 0.5% of the lung zone. **(c)** Image shows mild centrilobular emphysema (arrows), which involved 0.5%–5.0% of the lung zone. **(d)** Image shows moderate centrilobular emphysema, which involved more than 5% of the lung zone. **(e)** Confluent emphysema. **(f)** Advanced destructive emphysema with vascular distortion.

**Figure 1e:**
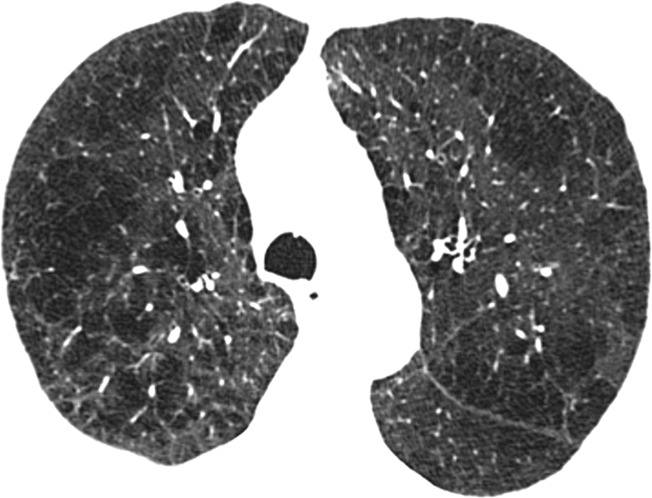
Axial CT images show severity grades of parenchymal emphysema. **(a)** Normal CT scan shows no emphysema. **(b)** Image shows trace centrilobular emphysema (circle), which involved less than 0.5% of the lung zone. **(c)** Image shows mild centrilobular emphysema (arrows), which involved 0.5%–5.0% of the lung zone. **(d)** Image shows moderate centrilobular emphysema, which involved more than 5% of the lung zone. **(e)** Confluent emphysema. **(f)** Advanced destructive emphysema with vascular distortion.

**Figure 1f:**
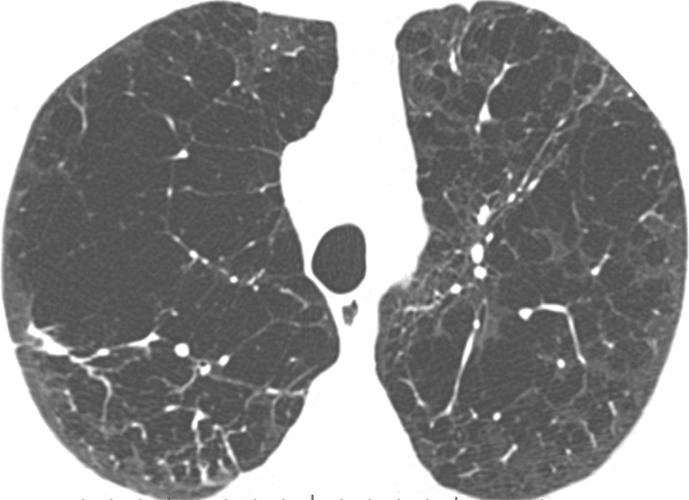
Axial CT images show severity grades of parenchymal emphysema. **(a)** Normal CT scan shows no emphysema. **(b)** Image shows trace centrilobular emphysema (circle), which involved less than 0.5% of the lung zone. **(c)** Image shows mild centrilobular emphysema (arrows), which involved 0.5%–5.0% of the lung zone. **(d)** Image shows moderate centrilobular emphysema, which involved more than 5% of the lung zone. **(e)** Confluent emphysema. **(f)** Advanced destructive emphysema with vascular distortion.

### Evaluation of Survival Times

Deaths were reported to our central study from the clinical centers. Sources included longitudinal follow-up contacts, reports from family members, obituaries and clinical records. We used information from the Social Security Death Index (SSDI) and the COPDGene longitudinal follow-up program to determine a survival or censoring time for each subject, taking care to avoid ascertainment bias, which can occur if death status is reported more consistently than alive status. Due to individual center institutional review board restrictions, 96% (3030 of 3171) of subjects had vital status searched by SSDI. Nine sites performed their own SSDI searches; all others used a centralized search performed by COPDGene staff. The median length of follow-up in this data set was 7.4 years (range, 30 days to 8.5 years). Further details of the survival analysis are provided in Appendix E1 (online).

### Statistical Evaluation

κ Statistics for the presence of emphysema and weighted κ statistics for grades of emphysema were calculated for each pair of analysts to assess interobserver agreement using “freq” procedure in SAS (SAS Institute, Cary, NC). Interobserver agreement was categorized as slight, fair, moderate, good, or excellent based on κ values of 0.20 or less, 0.21–0.40, 0.41–0.60, 0.61–0.80, and 0.81 or higher, respectively (27).

Descriptive statistics of baseline characteristics were calculated and compared between grades of parenchymal emphysema. Overall F-tests from analysis of variance models were used to compare continuous characteristics between grades using the “GLM” procedure in SAS (version 9.3); categoric characteristics were compared between grades using χ^2^ tests in the SAS “Freq” procedure.

The hazard of death was compared between parenchymal emphysema grades using a shared frailty model, an extension of the Cox proportional hazards model that can account for heterogeneity among study sites (28). First, a base model was fit including emphysema grade (categoric) as the primary explanatory variable, while controlling for age, weight, height, race (non-Hispanic White vs African American), pack-years of smoking, current smoking status (yes or no), and education level (some college vs high school or less). A normally distributed random effect was included as a linear predictor to account for correlation in the data due to clustering of subjects by study site. LAA-950 and FEV_1_ were added to this base model separately and then together to determine if emphysema grade was associated with survival, independent of quantitative CT measures of emphysema and spirometric measures of lung function at baseline. As sensitivity analyses, Cox proportional hazards models including study site as a fixed effect and Cox models accounting for correlation using robust sandwich covariance matrix estimates were also fit and produced similar results (29,30). All survival models were fit using the “phreg” procedure in SAS, version 9.3.

## Results

A total of four trained research analysts performed the readings for our study, with two readings per CT examination. Observer agreement among the analysts is shown in Table 1. κ Values and weighted κ values for presence and grade of emphysema were all good to excellent.

**Table 1:**
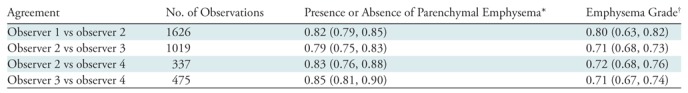
Observer Agreement for Visual CT Features

*Data are κ values, with 95% confidence intervals in parentheses.

^†^Data are κvalues, with weighted 95% confidence intervals in parentheses.

No evidence of emphysema was found in 1082 of the 3171 subjects (34%); a similar proportion (35%) had either trace or mild emphysema. Moderate emphysema was seen in 15%, confluent emphysema in 11%, and advanced destructive emphysema in 4% (Table 2). Compared with subjects with no or mild emphysema, subjects with advanced grades of emphysema were relatively older, were more likely to be non-Hispanic Whites than African-Americans, had a lower BMI, and had a relatively higher tobacco exposure, but were less likely to be current smokers. There was no consistent sex difference. Increasing severity of parenchymal emphysema was associated with progressively increasing airflow obstruction and decreasing 6 minute walk distance, as well as increasing severity of dyspnea measured by MMRC score. Notably, some degree of parenchymal emphysema was found in 562 (44%) of 1285 subjects with no spirometric abnormality (GOLD 0), and in 162 (52%) of 312 PRISm subjects (*P* = .011 for difference between GOLD 0 and PRISm). The prevalence of emphysema increased dramatically with GOLD stage, being found in 200 of 266 subjects with GOLD stage 1 COPD (75%), 537 of 655 subjects with GOLD stage 2 (82%), 388 of 408 subjects with GOLD stage 3 (95%), and 221 of 223 subjects with GOLD stage 4 (99%). With increasing emphysema severity along the Fleischner scoring scale, there was a clear and consistent pattern of increasing severity of airflow obstruction (decreasing FEV_1_ and FEV_1_/FVC ratio) and increased respiratory symptoms (as measured by SGRQ score and MMRC dyspnea score). Severity grading of emphysema also rose with increasing GOLD stage.

**Table 2:**
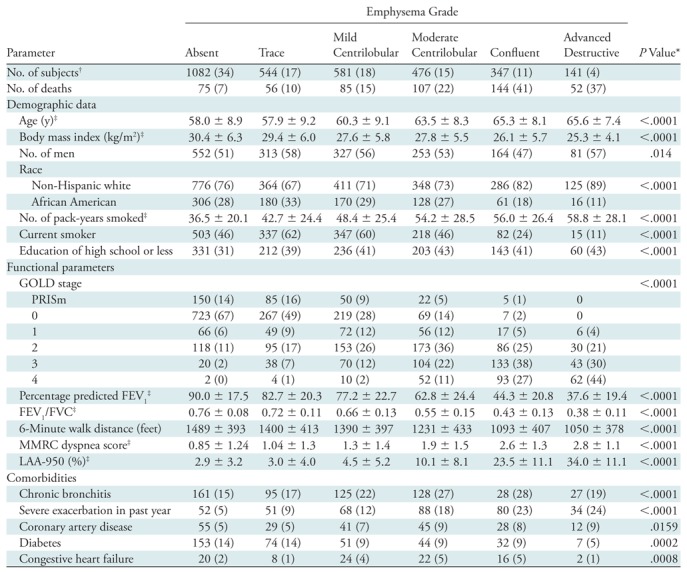
Mortality, Demographics, Functional Parameters, and Comorbidities according to Visual Grade of Emphysema

Note.—Unless otherwise specified, data are numbers of subjects, with percentages according to emphysema grade in parentheses.

*P value for differences across emphysema grades, calculated with χ^2^ test for categoric variables and with *F* test from analysis of variance for continuous variables.

^†^Percentages are according to total number of subjects.

^‡^Data are means ± standard deviations.

**Figure 2:**
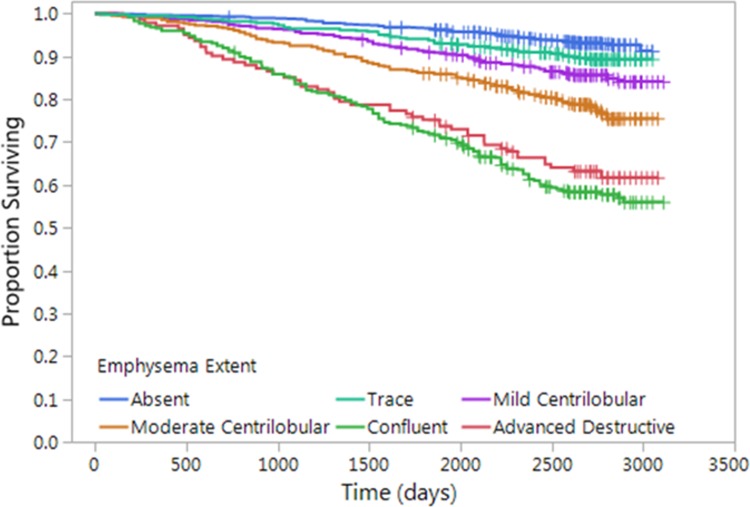
Graph shows relationship between parenchymal emphysema pattern and survival. Kaplan-Meier curves show decreasing survival with increasing grade of emphysema severity.

There were 519 deaths in the cohort. Kaplan-Meier analysis (Fig 2) showed decreasing survival with increasing grade of emphysema severity. On multivariable analysis, adjusted for race, sex, age, weight, height, smoking pack-years, current smoking status, and educational level (Table 3, model 1), every visual grade of emphysema (except for trace emphysema) was associated with a striking increase in mortality, with estimated hazard ratios of 1.7 for mild CLE (95% confidence interval [CI]: 1.2, 2.4), 2.5 for moderate CLE (95% CI: 1.8, 3.4), 5.0 for confluent emphysema (95% CI: 3.7, 6.8), and 4.1 for advanced destructive emphysema (95% CI: 2.8, 6.1). The mortality associations for mild CLE, moderate CLE, and confluent emphysema persisted after adjustment for quantitative measures of severity of emphysema (Table 3, model 2). After adjustment for FEV_1_ (model 3), the mortality risk of moderate, confluent, and advanced destructive emphysema persisted. After adjustment for BODE index (model 4), the increased risk of moderate and confluent emphysema persisted, and after adjustment for both LAA-950 and for BODE index (model 5), the increased risk of moderate and confluent emphysema persisted. The full Cox proportional hazards models are presented in Table E2 (online).

**Table 3:**
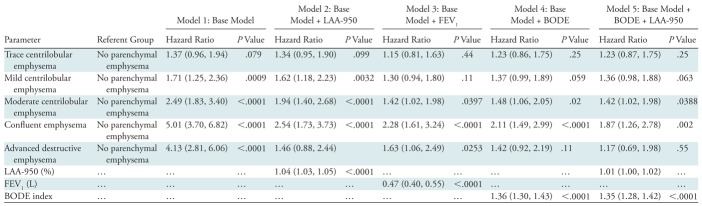
Cox Multivariable Models for Predicting Mortality

Note.—Models are adjusted for age, race, sex, weight, height, smoking pack-years, current smoking status at enrollment, and educational level. The full model is presented in Appendix E1 (online). Data in parentheses are 95% confidence intervals.

## Discussion

In this study, we used visually characterized patterns of emphysema in a large population (*n* = 3171) of current and former smokers using the Fleischner Society classification system. Visual classification of emphysema pattern was an independent predictor of mortality. We showed that the Fleischner classification patterns can be applied by trained research analysts with good to excellent interobserver agreement. Emphysema was identified in 66% of subjects, increasing in prevalence with increasing GOLD stage. However, we also found emphysema in a high proportion (44%) of subjects without spirometric impairment, and an even higher prevalence (52%) (*P* = .011) in the PRISm group, who have reduced FEV_1_ but preserved FEV_1_/FVC ratio. Most notably, the presence of any visual grade of emphysema (beyond trace) was associated with significantly increased mortality, and this increased mortality persisted after adjusting for quantitative severity of emphysema (LAA-950), except among those with advanced destructive emphysema. The mortality effect persisted for some grades of emphysema after adjusting for FEV_1_ and for BODE index, both of which are established risk predictors for mortality. These results suggest that visual scoring of thoracic CTs provides independent prognostic information for the clinical management of ever-smokers.

Our results extend previous studies on the relationship between emphysema subtypes and disease severity in cigarette smokers, which were performed and published prior to the implementation of the Fleischner Society classification. Previous studies generally classified emphysema as CLE, panlobular emphysema, and paraseptal emphysema (16,31–33). Because true histologic panlobular emphysema is uncommon in smoking-related emphysema, the Fleischner classification uses the terms “confluent emphysema” and “advanced destructive emphysema” in place of what would previously have been collectively called panlobular emphysema (12). The five-point Fleischner grading system offers the possibility to more precisely grade the visual severity of parenchymal emphysema. Our study shows a clear gradient of worsened airflow obstruction and greater respiratory symptoms with increasing emphysema grade, supporting the Fleischner scoring scale as a valid discriminatory tool to assess emphysema severity.

The current results agree with a study of 318 smokers from the Multi-Ethnic Study of Atherosclerosis (MESA) (16), which found that patients with either CLE or panlobular emphysema had greater dyspnea, reduced walk distance, and lower diffusing capacity than those without emphysema, while those with panlobular emphysema had reduced body mass index. Similarly, we found that subjects with confluent or advanced destructive emphysema (likely equivalent to panlobular emphysema in their study) had lower BMI than those with mild CLE.

These findings are also congruent with studies showing that extent of emphysema measured by quantitative CT is associated with increased mortality. A study of 947 ever-smokers found a 19-month shorter adjusted median survival in subjects with medium or high levels of emphysema by quantitative assessment relative to those with low levels of emphysema (4). In a study of 6814 MESA participants, the presence of emphysema defined by quantitative evaluation was strongly associated with increased mortality, even in those without traditional risk factors (3). Our study confirms the mortality effect associated with quantitative measurement of emphysema and additionally identifies an independent mortality effect from visually detected emphysema. Notably, this independent association with increased mortality was seen even for mild CLE (hazard ratio of 1.7 (95% CI: 1.2, 2.4) compared with no emphysema, remaining essentially the same after adjustment for quantitative emphysema severity). It is interesting that the adjusted mortality was lower in the subjects with advanced destructive emphysema than in those with confluent emphysema; the reason for this is unclear.

Importantly, our findings suggest that visual analysis of emphysema patterns provides mortality information that is independent of, and complementary to, quantitation of LAA-950. Discordance between visual and quantitative detection of emphysema has been shown (31); this discordance should not be surprising, as quantitative evaluation using LAA-950 or other methods provides a relatively crude global index of lung density that can be affected by image noise, and may not detect mild or localized emphysema. Because visual emphysema grading is less sensitive to image noise, it more precisely discriminates between subjects with and without emphysema. It will be helpful and important to compare the visual measures with more sophisticated quantitative methods (34).

Defining the mechanisms for increased mortality in subjects with emphysema will require further evaluation, including adjudication of cause-specific mortality (now underway in the COPDGene cohort). In severe emphysema, increased mortality likely relates at least in part to respiratory deaths. However, this possibility seems less likely in subjects with mild or moderate CLE, in whom percentage predicted FEV_1_ was relatively preserved. One alternative is lung cancer, since several studies have shown increased risk of lung cancer for visually identified emphysema (35,36), but not for quantitative emphysema assessment (37–39). This disparity suggests that visual emphysema is a superior marker of smoking-related injury to the lung, relative to current quantitative algorithms. It is also possible that the increased mortality is due to an increased incidence of cardiovascular events (40).

A noteworthy feature of our study is the high interobserver agreement, equal to or better than that found in previous studies involving trained radiologists (16,31). We attribute the low observer variation to the use of a progressive training model, with double reads for all CT examinations. We acknowledge that visual analysis is subjective, and requires substantial training. It is unrealistic to expect research analysts to provide readings for clinical scans. Nevertheless, it seems reasonable to expect that, after appropriate training with online reference standards, the five-point classification system for parenchymal emphysema can potentially be incorporated into routine readings of thoracic CT scans (including lung cancer screening scans) by radiologists who do not have access to quantitative imaging.

A limitation of our study was the exclusion of approximately 20% of our original study population because of missing or suboptimal CT or because survival information was not available. However, the excluded subjects had similar levels of physiologic and spirometric impairment to the included group. A second limitation is the proportion of our subjects with COPD (49%), higher than would be expected in an unselected population of cigarette smokers. Nevertheless, the magnitude and consistency of the mortality differences identified across the spectrum of emphysema severity suggest that these results should be applicable to the broader population. We did not evaluate the additional effects of nongated coronary artery calcium scores on all-cause mortality and major adverse cardiac events; this will certainly be the topic of further study.

We conclude that the Fleischner Society classification provides a valid, reproducible index of emphysema severity that is associated with both physiologic impairment and mortality risk. Applying this system to routine clinical radiology readings could identify individuals at higher risk of death, potentially leading to preventive interventions, including smoking cessation and other risk-factor modifications.

**Summary**

The Fleischner Society classification of emphysema provides a valid, reproducible index of emphysema severity that is associated with both physiologic impairment and mortality risk.

**Implications for Patient Care**

■ Application of the Fleischner Society visual classification of emphysema provides a reproducible index of disease severity.
■ Routine use of the Fleischner Society classification of emphysema could identify individuals at higher risk of death, potentially leading to preventive interventions, including smoking cessation and other risk factor modifications.

## APPENDIX

Appendix E1; Tables E1–E2 (PDF)
