# Supplementary material for: CT-based Visual Classification of Emphysema: Association with Mortality in the COPDGene Study
Source: Radiology. 2018 May 15;288(3):859–66. doi: 10.1148/radiol.2018172294 (PMC6122195; doi:10.1148/radiol.2018172294)

©RSNA, 2018
10.1148/radiol.2018172294

Appendix E1

Methods

Visual Analysis

To develop a consensus dataset for reference and training, five Fleischner Society members performed visual evaluation of 700 CT scans from the COPDGene cohort, using the previously described classification system (12). These 700 scans included 100 subjects randomly selected from each of the following groups: nonsmoking controls, smokers without COPD, smokers with GOLD stages 1, 2, 3, and 4 COPD, and smokers with FEV1 < 80% predicted, but with FEV1/FVC ratio ≥ 0.7, defined as "Preserved Ratio Impaired Spirometry" (PRISm) (23,24).

The Fleischner member consensus set of 700 CT scans formed the training set for visual CT readings by research analysts (who did not have previous training on reading CT images). A continuous learning method was applied (41). Each analyst in training scored a set of 20 training CTs using a Powerpoint visual reference guide; then the scores were compared with the Fleischner Society standard scores, and discordant scans were reviewed by the analyst. The images were viewed at standard window settings (window width 1500 and window level-700 for airways, and window width 700 and window level-750 for emphysema). A further 80 scans were similarly scored in batches of 20, with review of all discordances after each batch. After this training, the analysts systematically scored scans in batches of 200. Each scan was read by two analysts, and discordances with regard to scoring were adjudicated by a thoracic radiologist. After each batch of 200, substantial discordances were again reviewed by the analysts.

Survival Analysis

Deaths and censoring times for those searched via SSDI were back censored three months from the last SSDI search date to account for the expected lag between a death and its appearance in the SSDI database. The last centralized SSDI search was performed on October 14, 2016. For sites performing their own searches, the last SSDI search date was consequently site-dependent.

For 141 subjects for whom an SSDI search could not be performed, additional mortality data could be ascertained from their participation in the longitudinal followup (LFU) program of COPDGene. To ensure these subjects were being actively followed over time, LFU participation required the regular submission of LFU surveys and completion of an LFU survey in the seven months prior to the death date or the date of dataset generation for those who were censored (December 18, 2016). Subjects with consistent follow-up were back censored six months prior to dataset generation date.

Reference

41. Sverzellati N, Devaraj A, Desai SR, Quigley M, Wells AU, Hansell DM. Method for minimizing observer variation for the quantitation of high-resolution computed tomographic signs of lung disease. J Comput Assist Tomogr 2011;35(5):596–601 .

Table E1: Comparison of Included and Excluded Subjects

	Included subjects	Excluded subjects	*P* value for difference
N	3171	829	
Demographics			
Age (mean + SD)	60 ± 9	58 ± 9	<0.0001
Body Mass Index (mean + SD)	29 ± 6	28 ± 6	0.096
Male sex (% of total)	1690 (53%)	492 (59%)	0.0018
Female sex (% of total)	1481 (47%)	337 (41%)	
Race (% of total)			
NonHispanic White	2310 (73%)	425 (51%)	<0.0001
Pack-years (mean + SD)	46 ± 26	42 ± 25	<0.0001
Current smoker (% of total)	1502 (47%)	493 (59%)	<0.0001
Education: High school or less (% of total)	1185 (37%)	355 (43%)	0.0041
Functional parameters			
GOLD Stage (% of total)			0.0002
PRISm	313 (10%)	117 (14%)	
0	1285 (41%)	356 (43%)	
1	266 (8%)	58 (7%)	
2	654 (21%)	137 (17%)	
3	408 (13%)	88 (11%)	
4	223 (7%)	61 (7%)	
FEV1% predicted (mean + SD)	75 ± 26	75 ± 26	0.061
FEV1/FVC (mean + SD)	0.6 ± 0.2	0.7 ± 0.2	0.0028
6-minute walk distance (mean + SD)	1356 ± 429	1207 ± 418	<0.0001
MMRC Dyspnea Score (mean + SD)	1.4 ± 1.5	1.4 ± 1.5	0.57
LAA-950% (mean + SD)	8 ± 11	6 9 ± 9	0.0011
Comorbidities			
Chronic Bronchitis (% of total)	634 (20%)	106 (13%)	0.17
Severe exacerbation in past year (% of total)	373 (12%)	106 (13%)	0.42
Coronary artery disease (% of total)	210 (7%)	44 (5%)	0.17
Diabetes (% of total)	361 (11%)	117 (14%)	0.031
Congestive Heart Failure (% of total)	92 (3%)	26 (3%)	0.72

Table E2: Full Cox Multivariable Models for Predicting Mortality

Parameter		Base model		Base model + LAA-950		Base model + FEV1		Base model + BODE		Base model + BODE+ LAA-950	
	Referent group	Hazard ratio (95% confidence limits)	*P*	Hazard ratio (95% confidence limits)	*P*	Hazard ratio (95% confidence limits)	*P*	Hazard ratio (95% confidence limits)	*P*	Hazard ratio (95% confidence limits)	*P*
Race	White vs African American	1.11 (0.87, 1.42)	0.41	1.08 (0.84, 1.38)	0.55	1.19 (0.93, 1.52)	0.17	1.05 (0.82, 1.34)	0.72	1.04 (0.81, 1.33)	0.76

Gender	Male vs Female	1.18 (0.92, 1.52)	0.19	1.12 (0.87, 1.43)	0.39	1.35 (1.06, 1.73)	0.0172	1.11 (0.88, 1.47)	0.0179	1.12 (0.87, 1.45)	0.37
Age (years)		1.04 (1.02, 1.05)	<0.0001	1.04 (1.02, 1.05)	<0.0001	1.02 (1.01, 1.03)	0.0034	1.04 (1.03, 1.05)	<0.0001	1.04 (1.02, 1.05)	<0.0001
Weight (kg)		1.00 (0.99, 1.00)	0.18	1.00 (1.00, 1.01)	0.67	1.00 (0.99, 1.00)	0.054	1.00 (0.99, 1.00)	0.056	1.00 (0.99, 1.00)	0.16
Height (cm)		1.00 (0.99, 1.02)	0.66	1.00 (0.98, 1.01)	0.75	1.02 (1.01, 1.04)	0.0017	1.01 (1.00, 1.02)	0.19	1.01 (0.99, 1.02)	0.27
Pack-years		1.01 (1.00, 1.01)	0.0018	1.01 (1.00, 1.01)	0.0011	1.00 (1.00, 1.01)	0.0235	1.01 (1.00, 1.01)	0.072	1.00 (1.00, 1.01)	0.055
Current smoking	Current vs former	1.21 (0.97, 1.50)	0.086	1.46 (1.16, 1.82)	0.0011	1.38 (1.11, 1.71)	0.0034	1.41 (1.14, 1.75)	0.0018	1.46 (1.17, 1.83)	0.0009
Education	Some college vs high school or less	0.85 (0.71, 1.02)	0.075	0.86 (0.72, 1.03)	0.11	0.92 (0.77, 1.11)	0.38	0.97 (0.80, 1.16)	0.71	0.96 (0.80, 1.16)	0.69
Trace centrilobular emphysema	No parenchymal emphysema	1.37 (0.96, 1.94)	0.079	1.34 (0.95, 1.90)	0.099	1.15 (0.81, 1.63)	0.44	1.23 (0.86, 1.75)	0.25	1.23 (0.87, 1.75)	0.25
Mild centrilobular emphysema	No parenchymal emphysema	1.71 (1.25, 2.36)	0.0009	1.62 (1.18, 2.23)	0.0032	1.30 (0.94, 1.80)	0.11	1.37 (0.99, 1.89)	0.059	1.36 (0.98, 1.88)	0.063
Moderate centrilobular emphysema	No parenchymal emphysema	2.49 (1.83, 3.40)	<0.0001	1.94 (1.40, 2.68)	<0.0001	1.42 (1.02, 1.98)	0.0397	1.48 (1.06, 2.05)	0.02	1.42 (1.02, 1.98)	0.0388
Confluent emphysema	No parenchymal emphysema	5.01 (3.70, 6.82)	<0.0001	2.54 (1.73, 3.73)	<0.0001	2.28 (1.61, 3.24)	<0.0001	2.11 (1.49, 2.99)	<0.0001	1.87 (1.26, 2.78)	0.002
Advanced destructive emphysema	No parenchymal emphysema	4.13 (2.81, 6.06)	<0.0001	1.46 (0.88, 2.44)	0.14	1.63 (1.06, 2.49)	0.0253	1.42 (0.92, 2.19)	0.11	1.17 (0.69, 1.98)	0.55
LAA-950 (%)				1.04 (1.03, 1.05)	<0.0001					1.01 (1.00, 1.02)	0.20
FEV1 (L)						0.47 (0.40, 0.55)	<0.0001				
BODE Index								1.36 (1.30, 1.43)	<0.0001	1.35 (1.28, 1.42)	<0.0001

Appendix E2

COPDGene Investigators–Core Units

Administrative Center: James D. Crapo, MD (PI); Edwin K. Silverman, MD, PhD (PI); Barry J. Make, MD; Elizabeth A. Regan, MD, PhD

Genetic Analysis Center: Terri Beaty, PhD; Ferdouse Begum, PhD; Robert Busch, MD; Peter J. Castaldi, MD, MSc; Michael Cho, MD; Dawn L. DeMeo, MD, MPH; Adel R. Boueiz, MD; Marilyn G. Foreman, MD, MS; Eitan Halper-Stromberg; Nadia N. Hansel, MD, MPH; Megan E. Hardin, MD; Lystra P. Hayden, MD, MMSc; Craig P. Hersh, MD, MPH; Jacqueline Hetmanski, MS, MPH; Brian D. Hobbs, MD; John E. Hokanson, MPH, PhD; Nan Laird, PhD; Christoph Lange, PhD; Sharon M. Lutz, PhD; Merry-Lynn McDonald, PhD; Margaret M. Parker, PhD;

Dandi Qiao, PhD; Elizabeth A. Regan, MD, PhD; Stephanie Santorico, PhD; Edwin K. Silverman, MD, PhD; Emily S. Wan, MD; Sungho Won

Imaging Center: Mustafa Al Qaisi, MD; Harvey O. Coxson, PhD; Teresa Gray; MeiLan K. Han, MD, MS; Eric A. Hoffman, PhD; Stephen Humphries, PhD; Francine L. Jacobson, MD, MPH; Philip F. Judy, PhD; Ella A. Kazerooni, MD; Alex Kluiber; David A. Lynch, MB; John D. Newell, Jr., MD; Elizabeth A. Regan, MD, PhD; James C. Ross, PhD; Raul San Jose Estepar, PhD; Jered Sieren; Berend C. Stoel, PhD; Juerg Tschirren, PhD; Edwin Van Beek, MD, PhD; Bram van Ginneken, PhD; Eva van Rikxoort, PhD; Jean-Paul Charbonnier; George Washko, MD; Carla G. Wilson, MS

PFT QA Center, Salt Lake City, UT: Robert Jensen, PhD

Data Coordinating Center and Biostatistics, National Jewish Health, Denver, CO: Douglas Everett, PhD; Jim Crooks, PhD; Camille Moore, PhD; Matt Strand, PhD; Carla G. Wilson, MS

Epidemiology Core, University of Colorado Anschutz Medical Campus, Aurora, CO: John E. Hokanson, MPH, PhD; John Hughes, PhD; Gregory Kinney, MPH, PhD; Sharon M. Lutz, PhD; Katherine Pratte, MSPH; Kendra A. Young, PhD

COPDGene Investigators–Clinical Centers

Ann Arbor, MI: Jeffrey L. Curtis, MD; Carlos H. Martinez, MD, MPH; Perry G. Pernicano, MD

Baylor College of Medicine, Houston, TX: Nicola Hanania, MD, MS; Philip Alapat, MD; Mustafa Atik, MD; Venkata Bandi, MD; Aladin Boriek, PhD; Kalpatha Guntupalli, MD; Elizabeth Guy, MD; Arun Nachiappan, MD; Amit Parulekar, MD

Brigham and Women's Hospital, Boston, MA: Dawn L. DeMeo, MD, MPH; Craig Hersh, MD, MPH; Francine L. Jacobson, MD, MPH; George Washko, MD

Columbia University, New York, NY: R. Graham Barr, MD, DrPH; John Austin, MD; Belinda D'Souza, MD; Gregory D.N. Pearson, MD; Anna Rozenshtein, MD, MPH, FACR; Byron Thomashow, MD

Duke University Medical Center, Durham, NC: Neil MacIntyre, Jr., MD; H. Page McAdams, MD; Lacey Washington, MD

HealthPartners Research Institute, Minneapolis, MN: Charlene McEvoy, MD, MPH; Joseph Tashjian, MD

Johns Hopkins University, Baltimore, MD: Robert Wise, MD; Robert Brown, MD; Nadia N. Hansel, MD, MPH; Karen Horton, MD; Allison Lambert, MD, MHS; Nirupama Putcha, MD, MHS

Los Angeles Biomedical Research Institute at Harbor UCLA Medical Center, Torrance, CA: Richard Casaburi, PhD, MD; Alessandra Adami, PhD; Matthew Budoff, MD; Hans Fischer, MD; Janos Porszasz, MD, PhD; Harry Rossiter, PhD; William Stringer, MD

Michael E. DeBakey VAMC, Houston, TX: Amir Sharafkhaneh, MD, PhD; Charlie Lan, DO

Minneapolis, MN: Christine Wendt, MD; Brian Bell, MD

Morehouse School of Medicine, Atlanta, GA: Marilyn G. Foreman, MD, MS; Eugene Berkowitz, MD, PhD; Gloria Westney, MD, MS

National Jewish Health, Denver, CO: Russell Bowler, MD, PhD; David A. Lynch, MB

Reliant Medical Group, Worcester, MA: Richard Rosiello, MD; David Pace, MD

Temple University, Philadelphia, PA: Gerard Criner, MD; David Ciccolella, MD; Francis Cordova, MD; Chandra Dass, MD; Gilbert D'Alonzo, DO; Parag Desai, MD; Michael Jacobs, PharmD; Steven Kelsen, MD, PhD; Victor Kim, MD; A. James Mamary, MD; Nathaniel Marchetti, DO; Aditi Satti, MD; Kartik Shenoy, MD; Robert M. Steiner, MD; Alex Swift, MD; Irene Swift, MD; Maria Elena Vega-Sanchez, MD

University of Alabama, Birmingham, AL: Mark Dransfield, MD; William Bailey, MD; Surya Bhatt, MD; Anand Iyer, MD; Hrudaya Nath, MD; J. Michael Wells, MD

University of California, San Diego, CA: Joe Ramsdell, MD; Paul Friedman, MD; Xavier Soler, MD, PhD; Andrew Yen, MD

University of Iowa, Iowa City, IA: Alejandro P. Comellas, MD; John Newell, Jr., MD; Brad Thompson, MD

University of Michigan, Ann Arbor, MI: MeiLan K. Han, MD, MS; Ella Kazerooni, MD; Carlos H. Martinez, MD, MPH

University of Minnesota, Minneapolis, MN: Joanne Billings, MD; Abbie Begnaud, MD; Tadashi Allen, MD

University of Pittsburgh, Pittsburgh, PA: Frank Sciurba, MD; Jessica Bon, MD; Divay Chandra, MD, MSc; Carl Fuhrman, MD; Joel Weissfeld, MD, MPH

University of Texas Health Science Center at San Antonio, San Antonio, TX: Antonio Anzueto, MD; Sandra Adams, MD; Diego Maselli-Caceres, MD; Mario E. Ruiz, MD

Figure E1
Consort diagram explaining study population

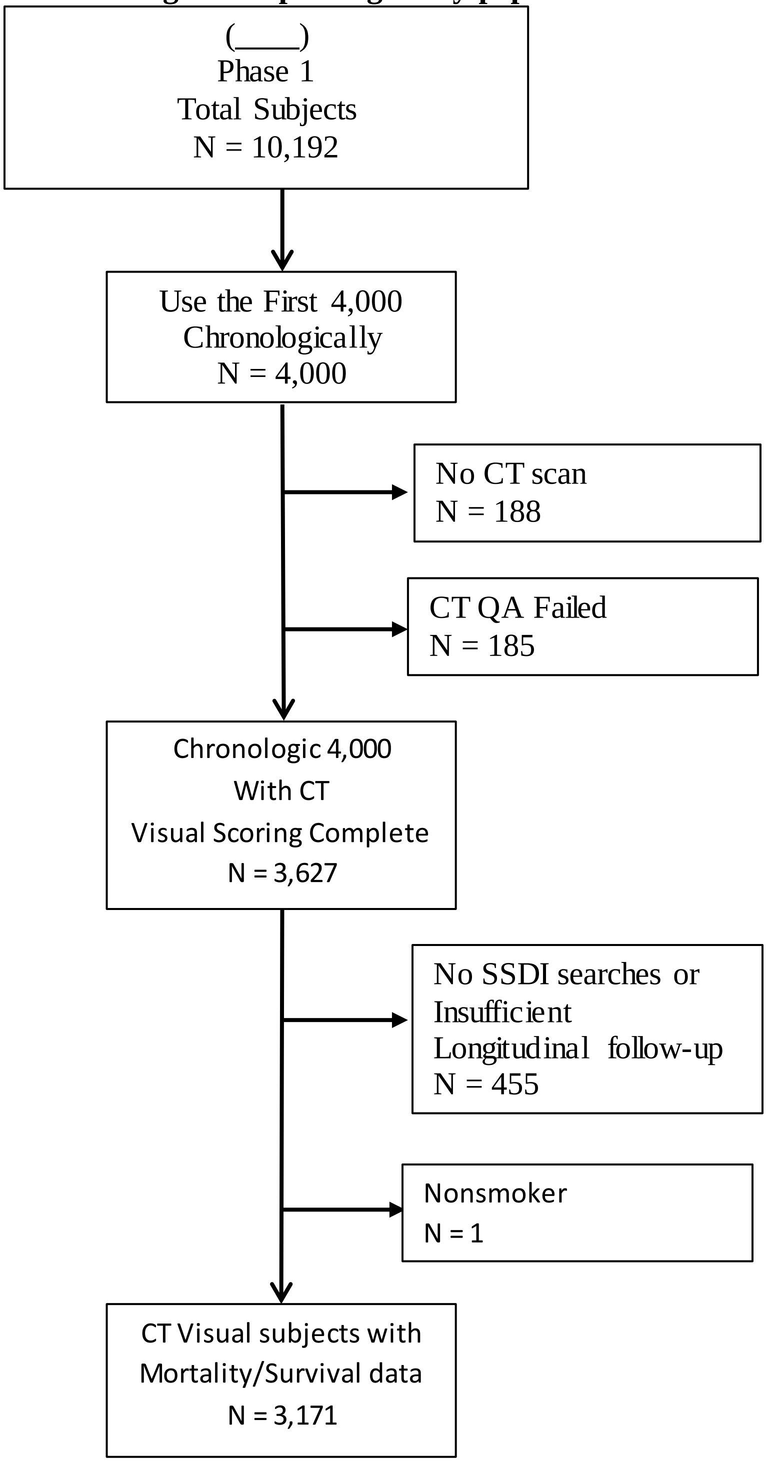

Supplement: Appendix E1; Tables E1–E2 (PDF) [file ry172294suppa1.pdf]
